# Absence of Mammary Development, and the Non-Secretion of Milk, in a Child-Bearing Female

**Published:** 1850-07

**Authors:** S. B. Hunt


*Absence of mammary development, and the non-secretion of milk, in a child-bearing female.*

EDITOR BUFFALO MEDICAL JOURNAL:—

*Dear Sir:* I send you an unusual case occurring in my practice nearly three years since. If you think it worth while you can publish it.

Mrs. A. N., confined in the summer of 1847, with her fifth child, has a chest as flat as a male's, with no evidence of the existence of any mammary gland. She has never given milk, and her children have been "brought up by hand;" three of them are now living. In her earlier confinements, efforts were made to procure the secretion, but failed, though I have no reason to doubt their being well directed. There has never been any swelling, pain, or other evidence of milk in the breasts. Her menses appear one month after confinement. She is robust, but not masculine in appearance.

Yours truly,

S. B. HUNT, M. D.

